# Effects of lasofoxifene and bazedoxifene on B cell development and function

**DOI:** 10.1002/iid3.37

**Published:** 2014-12-02

**Authors:** Angelina I Bernardi, Annica Andersson, Louise Grahnemo, Merja Nurkkala-Karlsson, Claes Ohlsson, Hans Carlsten, Ulrika Islander

**Affiliations:** 1 Centre for Bone and Arthritis Research, Department of Rheumatology and Inflammation Research, The Sahlgrenska Academy, University of GothenburgSweden; 2 Centre for Bone and Arthritis Research, Department of Internal Medicine and Clinical Nutrition, The Sahlgrenska Academy, University of GothenburgSweden

**Keywords:** Antibody production, B cell development, estrogen, mouse, SERMs

## Abstract

The third generation selective estrogen receptor modulators lasofoxifene (las) and bazedoxifene (bza) are indicated for treatment of postmenopausal osteoporosis. 17β-Estradiol (E2) and the second generation SERM raloxifene (ral) have major effects on the immune system, particularly on B cells. Treatment with E2 or ral inhibits B lymphopoiesis and treatment with E2, but not ral, stimulates antibody production. The effects of las and bza on the immune system have not been studied. Therefore, the aim of this study was to investigate their role in B cell development, maturation, and function. C57BL/6 mice were sham-operated or ovariectomized (ovx) and treated with vehicle, E2, ral, las, or bza. All substances increased total bone mineral density in ovx mice, as measured by peripheral quantitative computed tomography. In uterus, bza alone lacked agonistic effect in ovx mice and even acted as an antagonist in sham mice. As expected, E2 decreased B cell numbers at all developmental stages from pre-BI cells (in bone marrow) to transitional 1 (T1) B cells (in spleen) and increased marginal zone (MZ) B cells as determined by flow cytometry. However, treatment with las or bza only decreased the last stages of bone marrow B cell development and splenic T1 B cells, but had no effect MZ B cells. E2 increased antibody-producing cells quantified by ELISPOT, but las or bza did not. In conclusion, las and bza differ from E2 by retaining normal number of cells at most B cell stages during B lymphopoiesis and maturation and by not increasing antibody-producing cells.

## Introduction

Women at menopause have a higher risk of developing osteoporosis 1, however hormone replacement therapy (HRT), containing estrogen and progestin, has been linked to an increased incidence of endometrial- and breast cancer 2. To maintain beneficial estrogenic effects while avoiding side effects, selective estrogen receptor modulators (SERMs) have been developed that can act as estrogen receptor agonists or antagonists depending on the tissue. Lasofoxifene (las) and bazedoxifene (bza) are third generation SERMs indicated for prevention and treatment of osteoporosis. They act as estrogen receptor agonists in bone and have minimal proliferative effect on the endometrium 3.

Apart from the effects on the female reproductive system and bone remodeling, estrogen influences the immune system in a diverse manner and has a complex role in autoimmunity. Estrogen deficiency caused by ovariectomy leads to an increase in B cell development 4. On the contrary, increased levels of estrogen due to pregnancy or estrogen treatment cause a reduction in bone marrow B lymphocytes 5–7, while the secretion of antibodies increases 8,9. B cells develop from hematopoietic stem cells in the bone marrow and the first cell type belonging to the B lineage is the pro-B cell. These cells differentiate in an IL-7 dependent manner into pre-BI cells 10, which go through proliferative expansion and enter the large pre-BII cell stage 11. After exiting the cell cycle, the cells become small pre-BII cells. Recombination of the immunoglobulin light chain results in the expression of a membrane-bound antibody and differentiation to immature B cells. Estrogen has been shown to inhibit the first step of B cell development; the transition from pro-B to pre-BI cells, resulting in a decrease in all subsequent B cell populations in the bone marrow 6,12. We have previously shown that, similarly to estrogen, the second generation SERM raloxifene (ral) reduces B cell precursors in both intact and ovariectomized (ovx) mice 9. In order to complete maturation, the immature B cells translocate from the bone marrow to the spleen where they as newly immigrants are called transitional 1 (T1) B cells. The T1 B cells then differentiate into transitional 2 (T2) B cells which mature into follicular (FO) B cells or marginal zone (MZ) B cells 13. The trophic B cell activating factor (BAFF) plays an important role in peripheral B cell homeostasis by determining the number of transitional cells that survive and by controlling the life span of mature cells 14,15. Estrogen has been shown to alter splenic B cell populations 16 with a prominent reduction in T1 B cells demonstrated in estrogen-treated ovx mice. In addition, estrogen increases MZ B cells 16 and BAFF expression in the spleens of ovx mice 17. After antigen encounter, MZ B cells and FO B cells can develop into short-lived plasma cells secreting antibodies of IgM isotype 18. In addition, FO B cells can enter germinal centers where class switch recombination occurs, giving rise to memory B cells or plasma cells, producing antibodies of IgG, IgA, or IgE isotypes 19. Treatment with estrogen, but not ral, leads to an elevated number of antibody-secreting plasma cells in bone marrow and spleen in ovx and sham-operated mice 8,9.

To our knowledge, the effects of third generation SERMs on the immune system have not been studied. Therefore, the aim of this study was to investigate how las and bza affect B cell development, maturation, and function. Sham and ovx mice were treated with vehicle, E2, ral, las, or bza and B cell populations in bone marrow and spleen were analyzed using flow cytometry. In addition, levels of BAFF were assessed with ELISA and antibody-secreting cells were quantified using ELISPOT.

## Materials and Methods

### Mice

This study was approved by the ethical committee for animal experiments in Gothenburg. Female C57BL/6N mice (Scanbur, NOVA-SCB AB, Sollentuna, Sweden) were kept in groups of 5–10 animal per cage under standard environmental conditions and fed soy-free chow and tap water ad libitum.

### Ovariectomy

Mice were sham-operated or ovariectomized (ovx) at the age of 8 weeks. Surgeries were performed under full anesthesia using Isoflurane (Baxter Healthcare Corporation, Chicago, IL, USA). Ovaries were removed through a midline incision of the skin followed by flank incisions of the peritoneum, and the skin was closed with metallic clips. Sham operations were carried out in a similar manner but without removal of ovaries. Carprofen (Rimadyl®, Orion Pharma Animal Health, Sollentuna, Sweden) was used as post-operative analgesia. Mice were allowed to rest for 10 days after surgery before initiation of treatment.

### Hormones and treatment

Mice received subcutaneous injections of 17β-estradiol-3-benzoate (E2; 1 µg/mouse/day, Sigma–Aldrich, St Louis, MO, USA), raloxifene (ral; 60 µg/mouse/day, Sigma–Aldrich), lasofoxifene (las; 4 µg/mouse/day, Pfizer, Inc., NY, USA) or bazedoxifene (bza; 24 µg/mouse/day, Pfizer) dissolved in 100 µL miglyol oil (Vendico Chemical AB, Malmo, Sweden). Bza and las were kind gifts from Pfizer. Control mice received miglyol oil only (100 µL/mouse/day). Mice were treated for 16 consecutive days. It has previously been shown that mice treated with estradiol in doses similar to the one used here obtain serum estradiol levels corresponding to low diestrus levels (10–25 pg/mL) 20. Body surface area calculations ensured that doses of ral, las, and bza used in mice were similar to those used in humans 21. Indeed, Table1 shows that the doses of ral, las, and bza used result in a significant increase of total bone mineral density (BMD) in ovx mice.

**Table 1:** Uterus weight and total BMD of mice after 16 days of hormone treatment

Operation	Treatment	Uterus weight (mg)	*P*-value veh vs. treatment	Total BMD (mg/cm^3^)	*P*-value veh vs. treatment
Sham	Veh	73 ± 12		458 ± 19	
	E2	169 ± 11	··	601 ± 17	··
	Ral	35 ± 1	0.083	485 ± 11	0.559
	Las	46 ± 2	0.314	508 ± 14	0.083
	Bza	30 ± 2	·	461 ± 12	1.000
OVX	Veh	10 ± 1		375 ± 7	
	E2	144 ± 3	··	547 ± 9	··
	Ral	20 ± 1	··	469 ± 10	··
	Las	29 ± 1	··	499 ± 11	··
	Bza	14 ± 1	0.159	443 ± 13	··

*n* = 9–10 mice/group. Data expressed as mean ± SEM.

· *P* < 0.05.

·· *P* < 0.001.

### Tissue collection and single cell preparation

At termination, mice were anesthetized with ketamine (Ketalar®, Pfizer) and medetomidine (Domitor®, Orion Pharma), bled, and killed by cervical dislocation. Uterus and spleen were dissected and weighted. The right femur was collected for assessment of BMD and the left femur for bone marrow preparation. Bone marrow cells were harvested by flushing the cavity of the femur with PBS using a syringe. Spleen tissue was mashed through a 70 µm nylon mesh filter. Spleen and bone marrow cells were centrifuged at 1500*g* for 5 min and re-suspended in Tris-buffered 0.83% NH_4_Cl solution (pH 7.4) to lyse erythrocytes. Cells were then washed in PBS, re-suspended in phenol red-free RPMI 1640 medium (PAA Laboratories, GE Healthcare, Uppsala, Sweden) and counted using an automated cell counter (Sysmex Europe GmbH, Nordenstedt, Germany).

### Flow cytometer analysis

Bone marrow cells (0.5 × 10^6^) were stained with B220-FITC (Becton Dickinson (BD) Pharmingen, Franklin Lakes, NJ, USA), IgM-PE (Southern Biotechnology Associates, Inc., Birmingham, AL, USA), c-kit (CD117)-APC (Biolegend, San Diego, CA, USA), CD25-APC (Biolegend), CD19-Brilliant Violet 421 (Biolegend), and CD93-PE-Cy7 (Biolegend). Splenocytes (0.5 × 10^6^) were stained with B220-FITC (BD Pharmingen), CD21-FITC (BD Bioscience), IgM-PE (Southern Biotechnology Associates, Inc.), CD93-APC (eBioscience, Vienna, Austria), CD19-Brilliant Violet 421 (Biolegend), and CD23-PE-Cy7 (eBioscience). All cells were analyzed in a FACS Canto II (BD) and data were further processed in Flow Jo version 10.0.4 (Three Star, Inc., Ashland, USA). All analyses started with a singlet gate, thereafter a lymphocyte gate and gates for indicated populations. Results are presented as absolute number of cells of the different populations.

### ELISPOT

The number of IgG, IgM, and IgA-secreting cells in bone marrow and spleen was assessed using the ELISPOT technique 22. Briefly, 96-well nitro-cellulose plates (Millipore, Billerica, MA, USA) were coated with F(ab)_2_ fragments of goat anti-mouse IgG, IgM, and IgA (Southern Biotechnology Associates, Inc.). After overnight incubation at 4°C and blocking with 5% fetal calf serum, 10^5^ or 10^6^ freshly isolated spleen or bone marrow cells in RPMI medium were added to each well. The plates were then incubated at 37°C in 5% CO_2_ and 95% humidity for 3.5 h followed by incubation with alkaline phosphatase-conjugated goat anti-mouse IgG, IgM, or IgA in a humidity chamber overnight. After rinsing with technical water, the substrate 5-bromo-4-chloro-3-indolyl phosphate (Sigmafast™ BCIP/NBT, Sigma–Aldrich) was added to the plates. The reaction was stopped by the addition of tap water and each well was microscopically examined for the appearance of dark blue spots. Number of Ig-secreting cells were expressed as the frequency of spot forming cells (SFC)/10^3^ B220^+^ cells. The number of B220^+^ cells was obtained using flow cytometry.

### ELISA

Serum BAFF was measured by the commercially available Quantikine ELISA (MBLYS0, R&D Systems, MN, USA) following the protocol provided by the manufacturer.

### Assessment of BMD

BMD was determined in femur by a peripheral quantitative computed tomography (pQCT) scan with Stratec pQCT XCT Research M software (version 5.4B; Norland, Fort Atkinson, WI, USA) at a resolution of 70 μm, as described previously 23. Total BMD was determined with a metaphyseal scan at the distal femur.

### Statistical analysis

Statistical analyses were performed using IBM SPSS software version 21 (IBM, Armonk, NY, USA). Results from sham and ovx groups were analyzed separately. Normality was graphically assessed. Experiments were terminated on different days; therefore variation between days was assessed and corrected for when needed using ANCOVA, otherwise ANOVA was used. All groups were compared with vehicle using Dunnet's post hoc test when equal variance was found, and Dunnet's T3 post hoc test when Levene's test revealed unequal variance. Data are presented as arithmetic mean + SEM. *P* values* < *0.05 were considered statistically significant.

## Results

### Effects of E2 and SERMs on uterus and bone

In order to determine the uteroproliferative effect of the different compounds, the uterine weight of sham and ovx mice was measured after treatment with E2 or SERM. In sham mice treatment with E2 increased the uterus weight compared with control mice. Ral and las did not differ from vehicle while treatment with bza decreased uterus weight in sham mice (Table1). In line with previous studies 9, treatment of ovx mice with E2 or ral resulted in increased uterus weight. This finding was also observed in las- but not bza-treated ovx mice (Table1).

The total BMD of the femur was determined by pQCT. In intact mice, only E2 increased total BMD while in ovx mice all treatments led to a significant increase in total BMD, confirming the physiological relevance of the utilized doses of the compounds (Table1).

### Las and bza suppress B lymphopoiesis at a later developmental stage compared to E2

**Figure 1:**
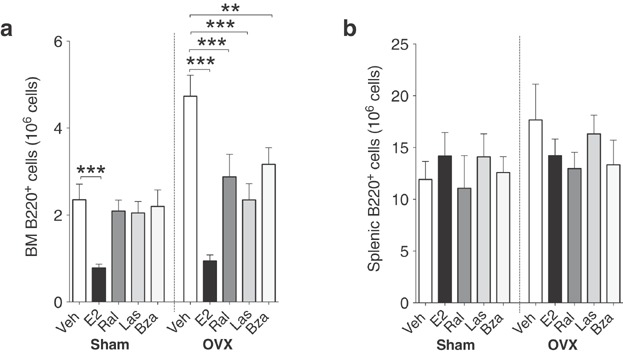
Lasofoxifene and bazedoxifene decrease number of B220^+^ cells in bone marrow of ovx mice. Total number of B220^+^ cells in (a) bone marrow and (b) spleen of sham (left) or ovx (right) mice treated with vehicle, E2 or SERMs, *n* = 9–10 mice/group. Data expressed as mean + SEM (**P < 0.01, ***P < 0.001).

Bone marrow cells were labeled with monoclonal antibodies against various B cell markers and analyzed using flow cytometry. As a pan-B cell marker, B220 (CD45R) was used. In sham mice, only E2 decreased the number of B220^+^ cells in the bone marrow (Fig. 1a, left). In ovx mice, E2, ral, las, and bza all reduced the number of B220^+^ cells (Fig. 1a, right). None of the treatments altered the number of B220^+^ cells in the spleen (Fig. 1b).

**Figure 2:**
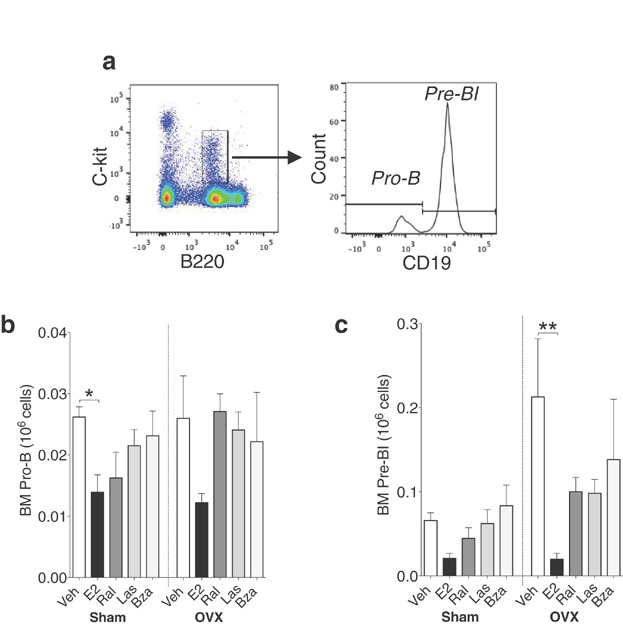
E2 decreases pre-BI cells in bone marrow of ovx mice. (a) Gating strategy for pro-B cells and pre-BI cells: B220^low^c-kit^+^ cells (left) were gated and further divided into CD19^−^ (pro-B cells) and CD19^+^ (pre-BI cells) (right). Number of (b) pro-B cells and (c) pre-BI cells of bone marrow lymphocytes in sham (left) or ovx (right) mice treated with vehicle, E2 or SERMs, *n* = 5–10 mice/group. Data expressed as mean + SEM (*P < 0.05, **P < 0.01).

Estrogen has an inhibitory effect on B cell differentiation and survival at the pro-B cell stage, preventing the transition to pre-B cells 6,12. In order to investigate how SERMs affect the transition between the two earliest stages of B cell development, the pro-B and pre-BI populations were identified by analyzing the expression of B220, c-kit and CD19. Both populations express B220 and c-kit (Fig. 2a, left). In order to divide the c-kit^+^ population into pro-B and pre-BI cells, CD19 was used as it is not yet expressed on pro-B cells, but up-regulated on pre-BI cells (Fig. 2a, right). None of the treatments altered the number of pro-B cells in sham or ovx mice (Fig. 2b). However, E2 decreased pre-BI cells with 90% in ovx mice compared with controls, while this population remained intact in SERM-treated mice (Fig. 2c, right). In sham-operated mice no changes were observed in the number of pre-BI cells (Fig. 2c, left).

**Figure 3:**
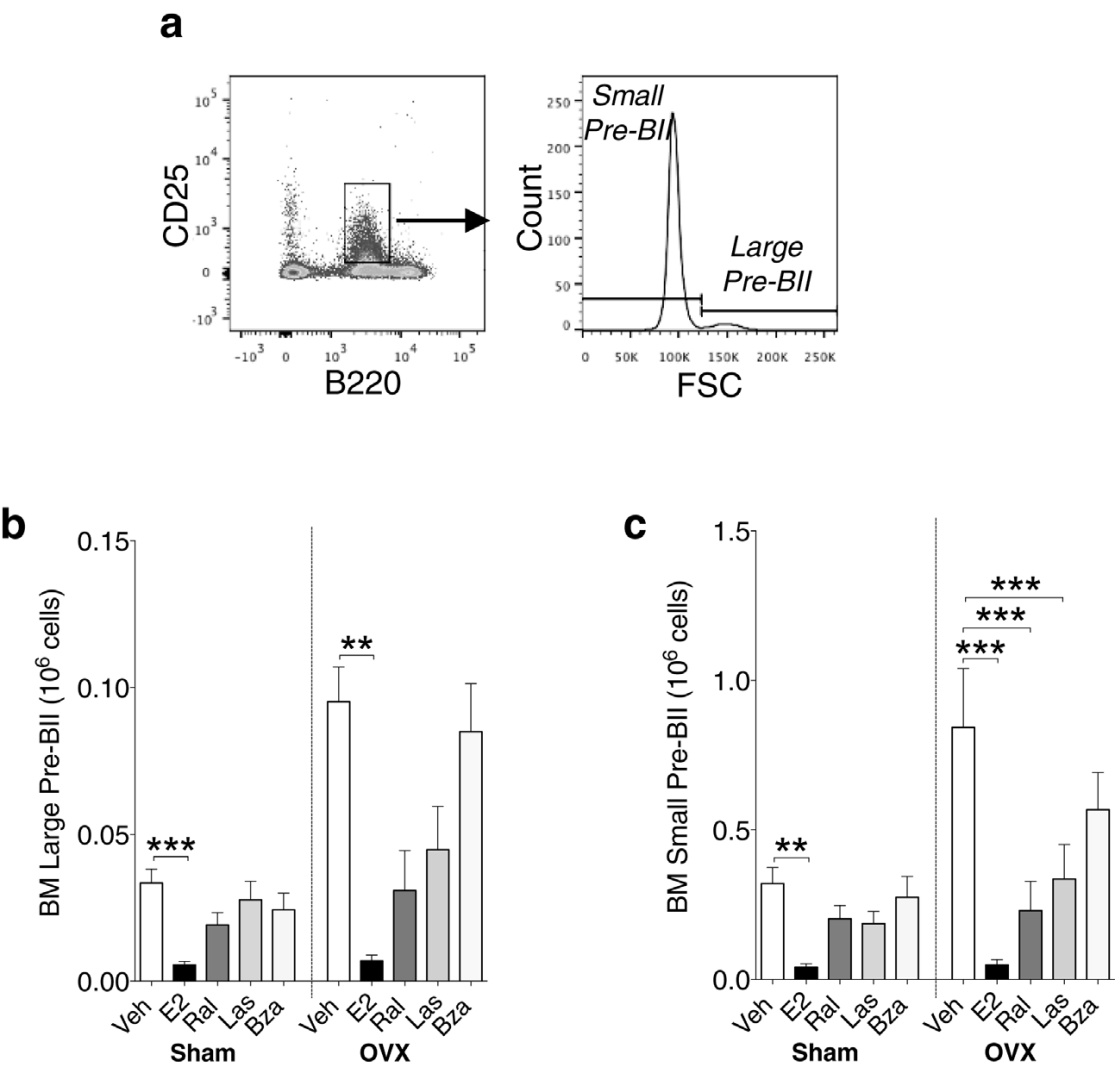
E2 decreases both large and small pre-BII cells, while lasofoxifene but not bazedoxifene decreases small pre-BII cells in bone marrow of ovx mice. (a) Gating strategy for large and small pre-BII cells: B220^low^CD25^+^ cells (left) were gated and further divided into small and large based on FSC (right). Number of (b) large pre-BII cells and (c) small pre-BII cells of bone marrow lymphocytes in sham (left) or ovx (right) mice treated with vehicle, E2 or SERMs, *n* = 5–10 mice/group. Data expressed as mean + SEM (**P < 0.01, ***P < 0.001).

Pre-BII cells (B220^+^, CD25^+^) (Fig. 3, left) were identified and divided into small and large pre-BII cells based on FSC (Fig. 3a, right). In both sham and ovx mice, E2 significantly reduced these populations (Fig. 3b and c). In ovx mice, treatment with ral and las induced a decrease in small pre-BII cells (Fig. 3c, right) while both populations remained unaffected by bza (Fig. 3b and c).

**Figure 4:**
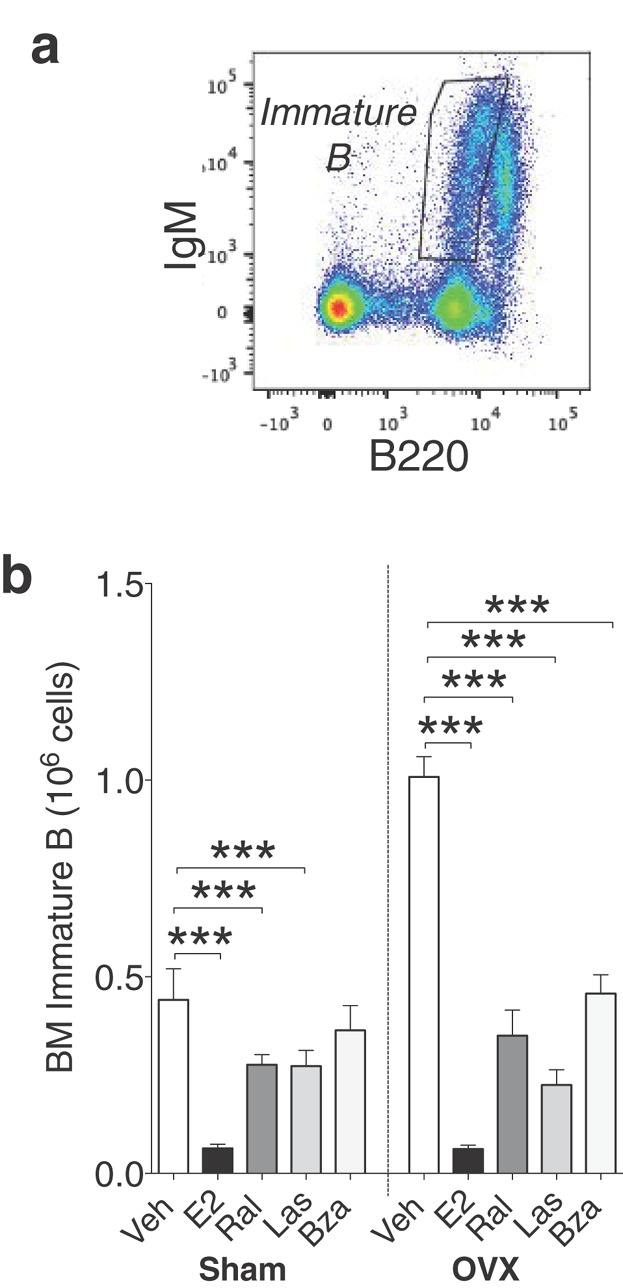
Lasofoxifene and bazedoxifene decrease immature B cells in bone marrow of ovx mice. (a) Gating strategy for immature B cells: B220^low^IgM^+^ cells were defined as immature B cells. (b) Number of immature B cells of bone marrow lymphocytes in sham (left) or ovx (right) mice treated with vehicle, E2 or SERMs, *n* = 9–10 mice/group. Data expressed as mean + SEM (***P < 0.001).

The subsequent developmental stage, the immature B cell population, was defined as B220^low^, IgM^+^ cells (Fig. 4a). In sham and ovx mice, E2 decreased this population (Fig. 4b). Interestingly, all three SERMs also strongly reduced the number of immature B cells in ovx animals (Fig. 4b, right).

### Las and bza decrease T1 cells but do not increase MZ B cells

**Figure 5:**
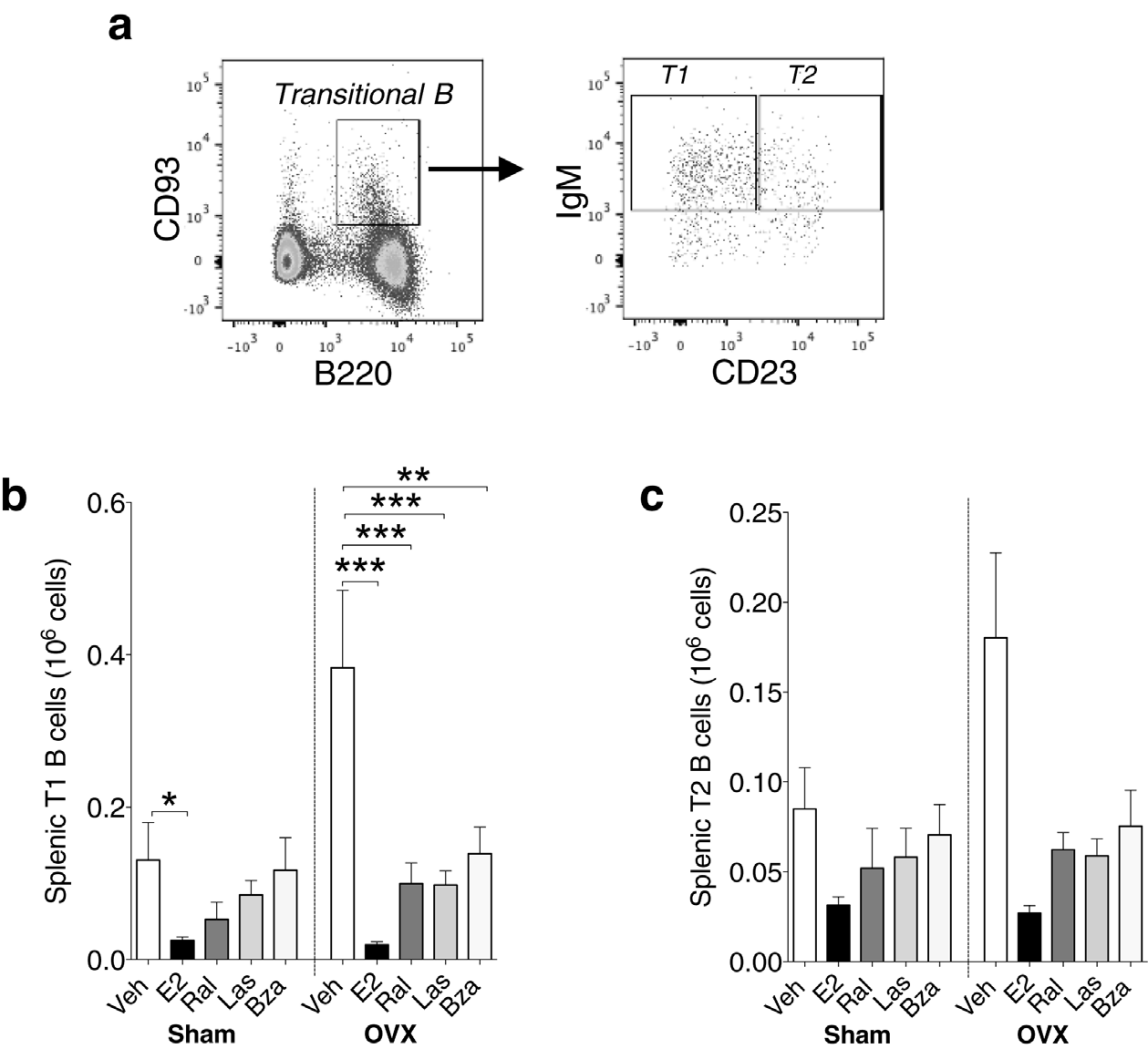
Lasofoxifene and bazedoxifene decrease T1 cells in the spleen of ovx mice. (a) Gating strategy for transitional B cells: CD93^+^ splenic lymphocytes were further divided into transitional 1 (T1) and transitional 2 (T2) B cells based on the expression of IgM and CD23. Number of (b) T1 B cells and (c) T2 B cells in spleen of sham (left) or ovx (right) mice treated with vehicle, E2 or SERMs, *n* = 9–10 mice/group. Data expressed as mean + SEM (*P < 0.05, **P < 0.01, ***P < 0.001).

Transitional 1 (T1) and transitional 2 (T2) cells were identified using antibodies against CD93, IgM, and CD23 (Fig. 5a). The number of T1 cells was reduced in ovx mice treated with E2, ral, las, or bza (Fig. 5b, right) while the number of T2 cells was not altered with any treatment (Fig. 5c, right). The number of T1 cells was reduced in sham mice after treatment with E2 while no changes were detected in the T2 fraction of B cells (Fig. 5b and c, left).

**Figure 6:**
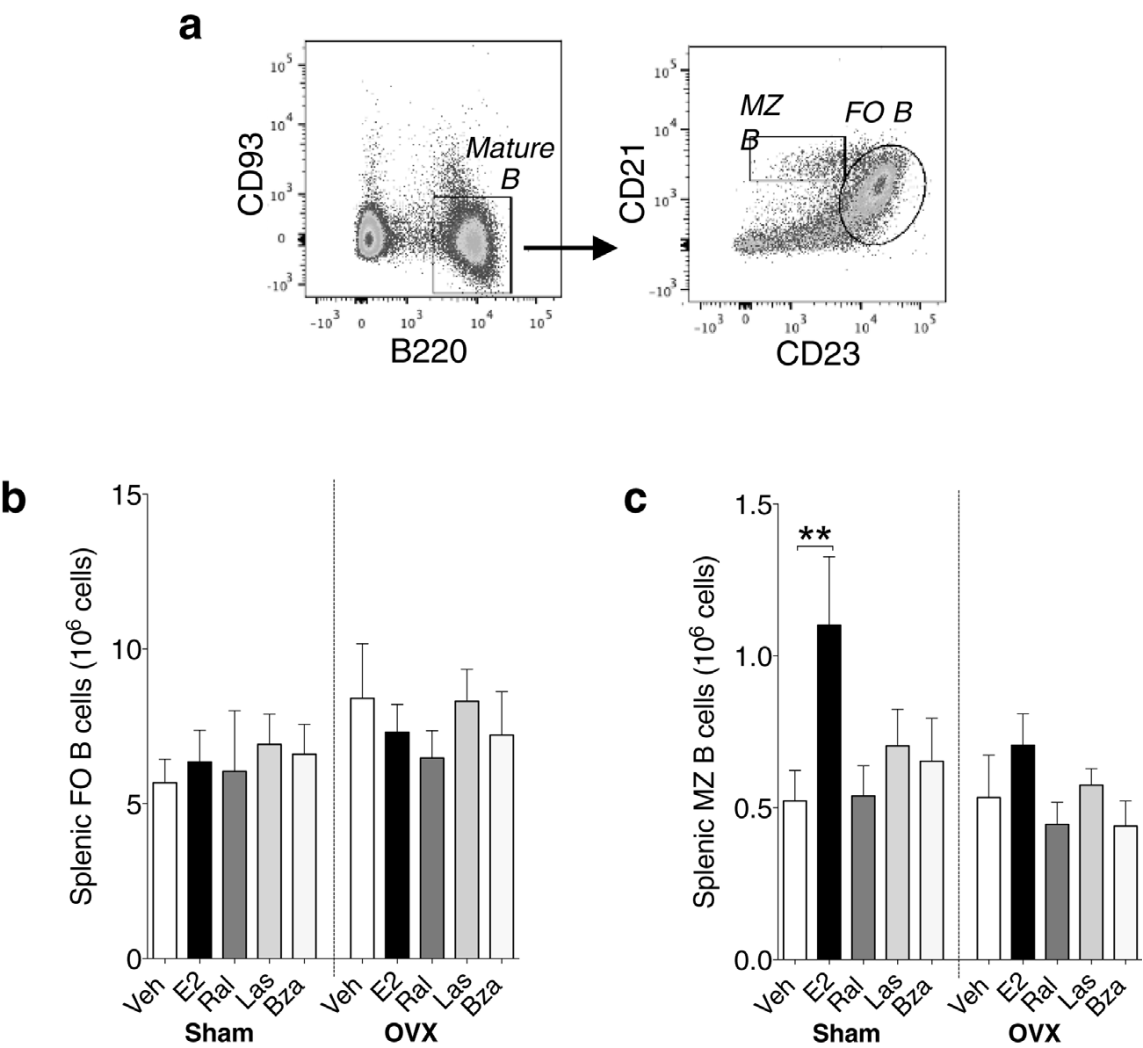
E2 but not lasofoxifene or bazedoxifene increases marginal zone (MZ) B cells the spleen. (a) Gating strategy for MZ B cells and follicular (FO) B cells in the spleen: Mature (CD93^−^) splenic lymphocytes were further divided into MZ B cells and FO B cells based on the expression of CD21 and CD23. Number of (b) MZ B cells and (c) FO B cells in spleen of sham (left) or ovx (right) mice treated with vehicle, E2 or SERMs, *n* = 5–10 mice/group. Data expressed as mean + SEM (**P < 0.01).

The expression of CD21 together with CD23 was analyzed on mature (CD93^−^) splenic lymphocytes to define follicular (FO) B cells and marginal zone (MZ) B cells (Fig. 6a). Consistent with previous studies 16 E2 did not have any impact on FO B cell population, but increased MZ B cells in sham mice (Fig. 6b and c). Conversely, none of the SERMs exerted any effect on FO or MZ B-cell populations (Fig. 6b and c).

### SERMs do not increase serum BAFF

**Figure 7:**
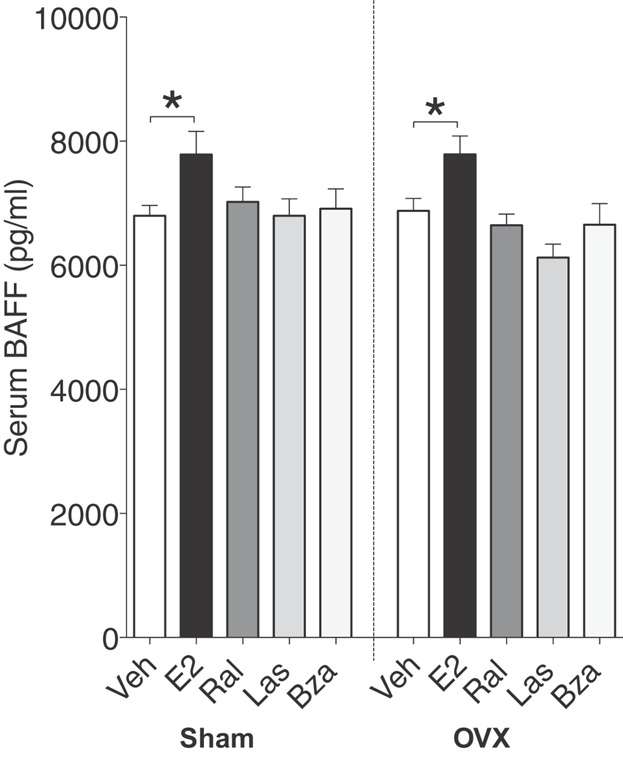
E2 but not lasofoxifene or bazedoxifene increases serum BAFF. BAFF was measured in serum of sham (left) or ovx (right) mice treated with vehicle, E2 or SERMs, *n* = 9–10 mice/group. Data expressed as mean + SEM (*P < 0.05).

Studies have demonstrated that in E2-treated ovx mice, the decrease in T1 cells is followed by an increase in BAFF 17. This increase is most probably a mechanism induced to correct for the reduction of T1 cells and restore peripheral B cell homeostasis 14. Since all SERMs decreased T1 B cells in ovx mice similarly to E2, the levels of BAFF in mice treated with SERMs were measured. An increase in serum BAFF was seen in E2-treated sham and ovx mice, but BAFF levels were not affected in mice treated with SERMs (Fig. 7).

### Las and bza do not influence antibody secretion

**Figure 8:**
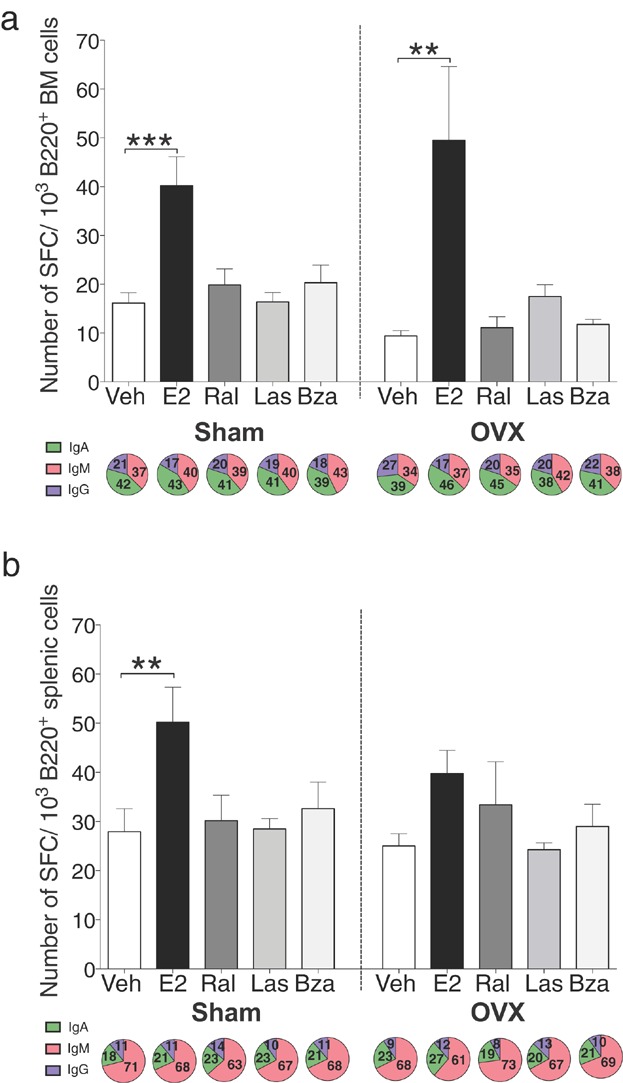
E2 but not lasofoxifene or bazedoxifene increases antibody-producing cells in bone marrow and spleen. Total number of spot-forming cells (SFC) of 10^3^ B220^+^cells (on top) and percentage producing IgA, IgM, and IgG (below) in (a) bone marrow and (b) spleen in sham (left) or ovx (right) mice treated with vehicle, E2 or SERMs, *n* = 9–10 mice/group. Data expressed as mean + SEM (**P < 0.01, ***P < 0.001).

It is known that estrogen enhances the activity of peripheral B cells, as demonstrated by an increased number of Ig-producing cells as well as an increased Ig production in estrogen-treated mice 8,9, while this effect has not been found after treatment with ral 9. In order to investigate how third generation SERMs influence B cell activity, the number of IgM, IgG, and IgA-producing cells in bone marrow and spleen were assessed. Treatment with E2 increased the total number of Ig-producing cells in both bone marrow (Fig. 8a) and spleen (Fig. 8b) of intact mice and in bone marrow of ovx mice (Fig. 8a). Ral and the third generation SERMs las and bza lacked the increasing effects on antibody-producing cells in both bone marrow and spleen (Fig. 8a and b). The proportions of cells producing the different Ig isotypes (IgA, IgM, IgG) were not altered with E2 or SERMs compared to vehicle in sham or ovx mice (Fig. 8a and b).

## Discussion

**Figure 9:**
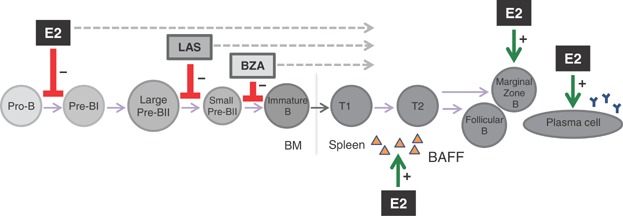
Schematic overview for the effects of E2, las, and bza on B cell development, maturation, and activity.

In this study the effects of the third generation SERMs las and bza on B cell development, maturation, and activity in sham and ovx mice were investigated. We here provide evidence that las and bza decrease immature B cells in the bone marrow and transitional 1 B cells in the spleen. In contrast to E2, the inhibition of bone marrow B cell development occurs at a later stage and thus fewer subpopulations are affected. Another difference compared with E2 was that no increase in serum BAFF levels or MZ B cells was seen with las or bza. Furthermore, neither las nor bza increase the number of antibody-secreting B cells in bone marrow or spleen. The effects of E2, las, and bza on B cell development, maturation, and activity are summarized in Figure 9. The present study also shows that bza lack the proliferative effect on uterus in ovx mice seen with E2 and other SERMs. Interestingly, bza decreases the uterus weight compared with vehicle in sham mice and thus acts as an antagonist in the uterus of intact mice.

It is known that the inhibitory effect of E2 on bone marrow B lymphopoiesis occurs at the transition from pro-B to pre-BI cells 6,12. In conjunction with this, our results show that E2-treated ovx mice have normal number of pro-B cells but decreased number of pre-BI cells, small and large pre-BII cells, and immature B cells. In contrast, neither las nor bza alter the number of pre-BI cells or large pre-BII cells. However, the number of small pre-BII cells was decreased with ral and las and the number of immature B cells was drastically reduced in ovx mice after all treatments.

The first part of B cell development depends on IL-7 secretion from stromal cells. Pro-B, pre-BI, and large pre-BII cells express IL-7 receptors that signal survival and proliferation 24. Without stromal cells that produce IL-7, the E2-mediated inhibition of the transition from pro-B to pre-BI cells does not occur, indicating that E2 inhibits B lymphopoiesis in an IL-7 dependent manner 6. However the IL-7-receptor is absent after the large pre-BII cell stage 25, and our results suggest that the inhibitory effect of las and bza occurs at the transition from large pre-BII (las) and small pre-BII (bza) to immature B cells. Therefore we propose that the las and bza-mediated inhibition of B lymphopoiesis occurs via an alternative, IL-7-independent, mechanism. The transition from small pre-BII cells to immature B cells involves the pairing of the immunoglobulin heavy chain and light chain, resulting in the cell surface expression of a B cell receptor (BCR). The BCR mediates survival and also promotes selection of B cells for further maturation 26. Therefore, the decrease in immature B cells seen in las- or bza-treated mice could be the result of altered BCR signaling at this stage. Furthermore, it has been shown that E2 reduces the mitotic activity of B lineage precursors, and the decrease in proliferation contributes to the subsequent reduction in bone marrow B cells 12. It is possible that also SERMs affect the mitosis of B cell precursors in the bone marrow, however further studies are needed to clarify the exact mechanism of the inhibitory effect of las and bza on B cells in the bone marrow.

In the spleen, it is known that E2 causes a reduction of transitional B cells 16. We found that also ral, las, and bza reduce the T1 B cells in ovx mice. Furthermore, survival of transitional B cells is regulated by BAFF 14 and the reduction in T1 B cells in E2-treated mice 16 has been shown to be accompanied by an increase in BAFF in spleen 17. Indeed we show an increase in serum BAFF after treatment with E2. This supports the idea that a decrease in transitional B cells induces BAFF production, to increase survival and maintain peripheral B cell homeostasis. Higher BAFF levels have been associated with an increase in the MZ B cell population 27 which indeed is the case in E2-treated mice 16. Interestingly, in mice treated with SERMs, the T1 B cell population was reduced, but BAFF levels were unchanged, followed by an unaltered MZ B cell population. We find this intriguing, raising the question why SERMs despite the prominent decrease in T1 cells, do not mediate a compensatory increase in BAFF to maintain peripheral B cell homeostasis. Furthermore, E2 has been shown to affect BCR signaling components; treatment with E2 up-regulates CD22 and SHP-1 in B cells, two negative regulators of BCR signaling. E2 has also been shown to reduce the phosphorylation of Erk1/2 after BCR activation in transitional cells and this reduction in BCR signaling contributes to the expansion of MZ B cells 17,28,29. In addition, E2 induces the anti-apoptotic protein Bcl-2 that leads to a resistance to BCR-mediated apoptosis in transitional B cells, also contributing to the increase in the MZ B cell population 28–30. Interestingly, the first generation SERM tamoxifene does not, in contrast to E2, induce an increase in CD22, SHP-1, or Bcl-2 in B cells 28. It is likely that also the third generation SERMs lack the decreasing effect on BCR signaling and the increasing effect on Bcl-2 expression, and that this together with the absent increase in BAFF can explain why las and bza do not increase MZ B cells.

It has been shown that the E2-mediated changes in bone marrow and spleen B cell populations are mediated by both subtypes of the estrogen receptor, ERα and ERβ 17,31,32. Las and bza bind with high affinity to both ERα and ERβ 33,34, however further studies are needed to clarify how the third generation SERMs regulate ER functions in different B cell subsets.

Estrogen, but not ral, increases Ig production and the number of Ig-producing cells in bone marrow and spleen 8,9. Our study confirms these observations and also shows that similarly to ral, las, and bza lack agonistic effect on antibody production (Fig. 9).

We found that sham mice generally are unaffected by the SERMs used in this study, suggesting that the endogenous estrogen in most cases retains normal levels of B cell subpopulations. However, from a clinical point of view, it is indeed relevant to study the effects on an estrogen-deficient system since las and bza are indicated as treatment for postmenopausal osteoporosis.

Estrogen has a complex role in autoimmunity. Estrogen treatment, HRT as well as pregnancy have been shown to ameliorate experimental collagen-induced arthritis (CIA) and rheumatoid arthritis (RA) 35–38. Ral reduces disease severity in ovx mice with CIA 37,39 and according to recent results, also las and bza show this effect. (Andersson, Bernardi et al., in preparation). The exact mechanisms for the alleviation of CIA after treatment with E2 or SERMs, and the relevance of effects on B cell development and function, remain to be clarified. In contrast to RA, systemic lupus erythematosus (SLE) is aggravated during pregnancy and HRT 38,40, and estrogen treatment worsens disease in mice with SLE-like systemic autoimmunity 41,42. The increase in antibody-production 8,9, and loss in B cell tolerance 30,43 induced by E2 treatment are possible mechanisms for how E2 exacerbate SLE. Interestingly, when mice with lupus-like disease were treated with ral and E2 simultaneously, ral restored B cell tolerance and improved disease 44. In addition, a recent clinical study showed that lumbar spine BMD improved, without change in disease activity, in postmenopausal SLE patients treated with ral 45. In this study, we show promising results that las and bza, similarly to ral, do not increase peripheral B cell activity. Additional studies will reveal if las and bza lack disease-aggravating effects in SLE. If so, the favorable safety profiles of third generation SERMs could motivate the use of these substances in the treatment of postmenopausal symptoms, as well as prevention and treatment of osteoporosis in SLE patients. Based on results presented in this study where we show that bza lack agonistic effect in the uterus of ovx mice and have antagonistic effect in the uterus of sham mice, we conclude that bza alone, or together with endogenous estrogen, is the SERM most suitable for further studies.
